# Prevention Strategies for Esophageal Cancer—An Expert Review

**DOI:** 10.3390/cancers13092183

**Published:** 2021-05-01

**Authors:** Elisa Marabotto, Gaia Pellegatta, Afscin Djahandideh Sheijani, Sebastiano Ziola, Patrizia Zentilin, Maria Giulia De Marzo, Edoardo Giovanni Giannini, Matteo Ghisa, Brigida Barberio, Marco Scarpa, Imerio Angriman, Matteo Fassan, Vincenzo Savarino, Edoardo Savarino

**Affiliations:** 1 Gastroenterology Unit, Department of Internal Medicine, University of Genoa, IRCCS Ospedale Policlinico San Martino, 16132 Genoa, Italy; emarabotto@libero.it (E.M.); djaf90@hotmail.it (A.D.S.); sebastianoziola@hotmail.it (S.Z.); pzentilin@unige.it (P.Z.); mgiulia.demarzo@gmail.com (M.G.D.M.); egiannini@unige.it (E.G.G.); vsavarin@unige.it (V.S.); 2 Digestive Endoscopy Unit, Department of Gastroenterology, IRCCS Humanitas Research Hospital, via Manzoni 56, 20089 Milan, Italy; gaia.pellegatta@humanitas.it; 3 Gastroenterology Unit, Department of Surgery, Oncology and Gastroenterology, University of Padova, 35128 Padova, Italy; matteoghisa@yahoo.it (M.G.); brigida.barberio@gmail.com (B.B.); 4 Clinica Chirurgica 1, Department of Surgery, Oncology and Gastroenterology, University of Padova, 35128 Padova, Italy; marcoscarpa73@yahoo.it (M.S.); imerio.angriman@unipd.it (I.A.); 5 Department of Medicine (DIMED), Surgical Pathology & Cytopathology Unit, University of Padua, 35121 Padua, Italy; matteo.fassan@gmail.com

**Keywords:** esophageal cancer, Barrett’s esophagus, endoscopy, screening, PPI, prophylaxis

## Abstract

**Simple Summary:**

In the last decades, there has been a rapid increase in the incidence and prevalence of esophageal cancer in many countries around the world. Although several important risk factors have been identified, strong evidence-based preventive strategies are still lacking, and the prognosis of patients diagnosed with esophageal cancer remains poor, with an average survival rate of 5 years for only 20%. This review is an attempt to summarize the epidemiology and risk factors of EC and to highlight the unresolved aspects of current prevention strategies in order to plan more fruitful future initiatives aimed at ameliorating the disappointing prognosis of this kind of digestive tumor.

**Abstract:**

In the last 30 years, we have witnessed a rapid increase in the incidence and prevalence of esophageal cancer in many countries around the word. However, despite advancements in diagnostic technologies, the early detection of this cancer is rare, and its prognosis remains poor, with only about 20% of these patients surviving for 5 years. The two major forms are the esophageal squamous cell carcinoma (ESCC), which is particularly frequent in the so-called Asian belt, and the esophageal adenocarcinoma (EAC), which prevails in Western populations. This review provides a summary of the epidemiological features and risk factors associated with these tumors. Moreover, a major focus is posed on reporting and highlighting the various preventing strategies proposed by the most important international scientific societies, particularly in high-risk populations, with the final aim of detecting these lesions as early as possible and therefore favoring their definite cure. Indeed, we have conducted analysis with attention to the current primary, secondary and tertiary prevention guidelines in both ESCC and EAC, attempting to emphasize unresolved research and clinical problems related to these topics in order to improve our diagnostic strategies and management.

## 1. Introduction

Over the past 30 years, we have witnessed a rapid increase in the incidence and prevalence of esophageal cancer (EC) in many countries around the world, and this tumor has become the most quickly increasing form of cancer in some populations [1]. Although some strong risk factors have been clearly identified, we remain in need of strong evidence-based preventive strategies. In fact, the prognosis of patients diagnosed with EC is still poor, and only a small percentage of them (about 20%) survive for 5 years.

This review has been carried out with the aim of summarizing the epidemiology, the risk factors and the various prevention strategies proposed for EC, attempting to highlight unresolved research problems related to these topics. To identify the relevant studies in these fields, a computerized (Medline) and manual literature search was performed for the period up to December 2020, with particular focus on the past 10 years. We used the following terms: “esophageal cancer”, “esophageal cancer epidemiology”, “esophageal squamous-cell carcinoma“, esophageal adenocarcinoma”, “esophageal cancer risk factors”, “esophageal cancer prognosis”, “esophageal cancer prevention”, “primary prevention”, “secondary prevention”, “tertiary prevention”, “esophageal squamous-cell prevention”, esophageal adenocarcinoma prevention”, “esophageal cancer prevention strategies”, “esophageal cancer chemoprevention”, “gastro-esophageal reflux disease”, “GERD”, “reflux complications”, “Barrett esophagus”, “esophageal low-grade dysplasia”, “esophageal high-grade dysplasia”, “esophageal early adenocarcinoma”, “proton pump inhibitors”, “PPIs”, “achalasia”, “Helicobacter pylori” “endoscopic screening”, “endoscopic surveillance”, “endoscopic therapy”, “anti-reflux surgery”, and “Barrett esophagus treatment”. We critically reviewed all full-text papers and relevant abstracts published in the English language. The reference lists from identified papers were searched to find any additional studies that could have been missed during the process.

## 2. Epidemiology

Esophageal cancer represents the eighth most common cancer worldwide and the sixth leading cause of cancer-related mortality [2]. In 2010, the United States experienced about 16,640 new cases and 14,500 deaths caused by EC. The GBD 2017 Esophageal Cancer Collaboration has published updated statistics on the global incidence and mortality of esophageal cancer as well as on the disability-adjusted life-years (DALYs) caused by the disease [3]. The authors have reported that there were 473,000 new cases of EC, 436,000 deaths due to EC, and 9.78 million DALYs caused by EC worldwide in 2017. The lifetime risk of developing this type of cancer is 0.8% for men and 0.3% for women, and, at present, the mean age of diagnosis is 67 years [4]. Moreover, approximately 80% of cases occur in developing countries [5] and the incidence of EC varies greatly with location [6]. For instance, at the national level, China had the highest number of incident cases (235,000) and deaths (213,000) in 2017, while the highest age-standardized incidence rates in the same year were observed in Malawi (23 cases per 100,000 population) and Mongolia (18.5 cases per 100,000) [3]. In contrast, Italy is one of the countries where EC is relatively rare; during the period 1998–2002, it represented 0.9% of all cancers among men and 0.3% among women and 1.9% of all cancer deaths among men and 0.8% among women [7]. In the area of the Italian Network of Cancer Registries (AIRT), the incidence of EC is 7.2 per 100,000 males/year and 2.1 per 100,000 females/year. Incidence rates vary remarkably across Italy, and the ratio between areas with the highest and the lowest rates is about 8:9. Generally, the highest rates are documented in the north-eastern areas and the lowest ones in the southern registries of this country. In the Veneto region [8], the risk of death due to EC is nearly twice as high as in the whole of Italy (5.7 versus 3.4 deaths × 100,000 population/year). As regards time trends, EC shows a decreasing time trend in both incidence and mortality. The decreased incidence of EC is mainly due to esophageal squamous-cell carcinoma (ESCC), while esophageal adenocarcinoma (EAC) is increasing. Nowadays, the frequency of EAC is one in every three ESCC [9].

EC has two main subtypes: ESCC and EAC, whereas other histological variants (such as esophageal melanomas, leiomyosarcomas, carcinoids and lymphomas) are rarely diagnosed [10]. ESCC accounts for about 80% of EC cases worldwide. In particular, in the so-called Asian belt, which includes Turkey, North-East Iran, Kazakhstan and northern and central China, the incidence of ESCC is extremely high, with more than one case per 1000 individuals annually [3,11].

During the last four decades, the incidence and the mortality rates associated to EAC have been rising, especially among white men, and in North America and Europe have exceeded ESCC, thus becoming the dominant histologic type of esophageal cancer (25.6 cases per million in 2006, annual increase 1.3% after 1996) [12,13]. Nevertheless, so far ESCC remains the predominant histological subtype of esophageal cancer in Asia, Southern and Eastern Africa, South America and among the African American population in North America.

Lastly, EC presents a dismal prognosis. In fact, survival rates for both histological subtypes are very low, with a five-year survival rate of 15% for EAC [14] and 15–25% for ESCC [10,15]. In order to achieve a major improvement in the prognosis of EC, preventive measures have been investigated, developed, and are highly warranted.

## 3. Risk Factors and Primary Prevention

The identification of risk factors is an important first step for primary prevention (Table 1).

### 3.1. Esophageal Squamous-Cell Carcinoma

Traditional risk factors for ESCC are tobacco use, alcohol consumption, genetic mutation of enzymes that metabolize alcohol, achalasia, caustic injuries and hot beverages, exposure to thoracic radiations, low socio-economic status, poor oral hygiene, nutritional deficiencies, and tylosis [16,17].

ESCC develops via a multistep process that begins with a normal squamous epithelium and progresses to low-grade intra-epithelial neoplasia (LGIN), high-grade intraepithelial neoplasia (HGIN) and ultimately into invasive carcinoma. Through this process, ESCC usually expands between the middle and the lower third of the esophagus.

In the reassessment of carcinogen factors for the esophagus, the International Agency for Research on Cancer included acetaldehyde from alcoholic beverages as a carcinogen of group 1 [18]. In fact, ingested alcohol is metabolized to acetaldehyde by alcohol dehydrogenase 1B, which is subsequently detoxified to acetic acid by aldehyde dehydrogenase 2 (ALDH2) [19]. Mutation in ALDH2 enzyme increases blood, salivary, and breath levels of acetaldehyde after alcohol intake [20,21], and this phenomenon is associated with increased risk of ESCC. Genetic polymorphism of this enzyme, which is prevalent among Mongoloids, but not in Caucasoid or Negroid populations, seems to account for the highest incidence of ESCC in Asian countries [22].

Additionally, direct contact between the carcinogenic substances contained in tobacco, such as nicotine-derived nitrosamine ketone, N1-nitrosonornicotine, polycyclic aromatic hydrocarbons, aromatic amines and carcinogen acetaldehyde, and the esophageal mucosa has been proven to increase the risk of ESCC [23]. In support of this theory, Hoffmann et al. reported that a large-scale population-based cohort study revealed that both past and current smokers have a higher risk of developing ESCC than individuals that have never smoked. Furthermore, among current smokers, the risk of ESCC increases in a dose-dependent manner.

To determine the positive association between alcohol use, tobacco use, and ESCC illustrated above, many case-control and cohort studies have been conducted in the past decades [24,25,26]. For example, a Japanese large-scale population-based cohort study showed that heavy alcohol consumption and cigarette smoking are strongly associated with ESCC, especially among heavy smokers with inactive allele of ALDH2, the so-called flushing response to alcohol [24], while smokers after an ethanol challenge had 7 times higher salivary acetaldehyde levels than non-smokers [27,28].

Other important risk factors are low levels of consumption of fruits and vegetables, deficiency of selenium, zinc or vitamin E. The risk of ESCC was found to be reduced by 31–35% for every 5-unit increased BMI [29]. It is somewhat surprising that obesity seems to exert a protective role on the development of ESCC, but this effect is far from being explained. Various potential biases on the evaluation of risk factors for ESCC have been considered, including smoking status, but none have enabled us to understand the reasons for this inverse relationship. On the contrary, the major documented risk of leanness for ESCC has been related to the fact that a poor diet leads to micronutrient deficiencies or malnutrition, but the protective influence of micronutrients for this cancer type is unknown.

Lastly, an increased risk of ESCC has been observed in patients with esophageal achalasia, a rare idiopathic motor disorder characterized by lack of relaxation of the lower esophageal sphincter and an absence of peristalsis. In these patients, the development of ESCC seems likely due to the chronic inflammation associated with the stasis of food in the esophagus and the production of nitrosamines by bacterial overgrowth [30]. Available data suggest that the estimated risk of ESCC in patients with achalasia is 11–50-fold greater than in the general population.

### 3.2. Esophageal Adenocarcinoma

The major risk factors for EAC include symptomatic gastro-esophageal reflux disease (GERD), Barrett’s esophagus, visceral abdominal obesity, tobacco use, assumption of drugs that relax the lower esophageal sphincter, as well as diet with a low intake of vegetables and fruit and rich in processed meat [31,32]. EAC is characterized by a multistep process that begins with a normal esophageal squamous epithelium and develops into columnar epithelium, low-grade dysplasia (LGD), high-grade dysplasia (HGD) and eventually EAC under the influence of acid and weakly acid exposure. About 75% of all EACs are localized in the distal tract of the esophagus [15].

A recent meta-analysis evaluated the association between symptoms of GERD and EAC. The results showed that patients presenting weekly and daily symptoms of GERD have, respectively, a 5-fold and a 7-fold increase in the probability of developing EAC, compared with individuals without symptoms or with less-frequent symptoms. Although the duration of symptoms was also associated with EAC, the results remain very heterogeneous and the thresholds unclear [33]. A pooled analysis from the international Barrett’s and Esophageal Adenocarcinoma Consortium (BEACON) has highlighted a strong relationship between gastroesophageal reflux (GER) exposure and esophageal adenocarcinoma, and moreover, a longer duration and increased frequency of reflux are both associated with higher carcinogenic risk [34]. Nevertheless, it is important to notice that up to 40% of all patients with EAC do not report GERD symptoms, and this has generated a heated debate among experts about the usefulness of endoscopic surveillance in patients with GERD [35].

One of the most dangerous complications of GERD is the development of Barrett’s esophagus (BE), defined as the replacement of squamous mucosa by columnar epithelium as a defense against exposure to acid and/or bile reflux. Recent studies that used 24-h-MII-pH monitoring showed that patients with BE have longer esophageal refluxate (both acid and weakly acidic) exposure time, a higher number of total reflux episodes, an increased number of re-reflux events, and prolonged acid and volume clearance times, compared with patients with erosive esophagitis (EE) and healthy volunteers [36,37]. Moreover, the number and proximal migration of reflux episodes are related to the extension of BE, whose length is also associated with the prospective risk of dysplasia with greater risk in longer segments [38]. However, only 10–15% of patients with chronic GERD develop BE, indicating that additional and unknown genetic and environmental factors are most certainly involved [39].

The estimated incidence of EAC in patients with BE is approximately 0.1–0.5% per year [40]. The risk is higher in patients with BE with low-grade (LG) and high-grade (HG) dysplasia, which degenerates into EAC in 1–10% per year and 40% per year, respectively [41]. A study published in 2011 demonstrated that the increasing severity of mucosal damage of GERD into EE and BE is associated with a progressively more severe deflection of esophageal manometric features, motility, and bolus transit pattern [42]. If this phenomenon is related, even just partially, to a severe esophageal mucosal inflammation induced by an increase of GER, which in turn induces a reduction in esophageal compliance and an increased resistance during bolus movement with a consequently delayed bolus transport, it could explain the development of BE on EE leading to EAC.

Moreover, obesity is associated with an increased risk of EAC by a factor that ranges between 2.4 and 2.8 [43]. In fact, patients with central obesity are more predisposed to hiatal hernia and present an increased intra-gastric pressure that enhances GER. Additionally, this population presents higher basal insulin and insulin-like-growth factor 1 (IGF-1) levels, which promote cell proliferation and determine cell differentiation [44]. These patients also present higher serum levels of leptin, a hormone secreted by visceral fat that potentially promotes carcinogenesis by mitogenic and angiogenic means [45]. A recent case-control study demonstrated that a large amount of visceral abdominal fat detected by a CT scan is associated with significant increase in the risk of BE [43]. In particular, this study shows how GERD may mediate some but not all of this association. As abdominal adiposity is more common in men, this may be a possible explanation for sex-related differences in cancer risk.

The association between Helicobacter pylori (Hp) infection and EAC is still debated. In fact, Hp infection induces atrophy of gastric mucosa and therefore decreases the acid production by oxyntic cells and the exposure of esophageal epithelium to acid, and this should in turn reduce the risk of BE and EAC. In this regard, a meta-analysis published in 2013 analyzed 15 observational studies and showed that the risk of EAC decreased by 41% among patients with Hp infection [46]. On the other hand, after initiating therapy and succeeding in the eradication of Hp, in most cases patients neither develop nor exacerbate GERD symptoms [47,48,49].

The main lifestyle and clinical factors known to influence the development of esophageal cancer and the related international recommendations are reported in Table 2.

## 4. Secondary Prevention

It is of fundamental importance to identify high-risk groups for ESCC and EAC in order to detect premalignant lesions early and offer them adequate monitoring. At present, upper endoscopy is the only tool utilized for secondary prevention through the direct visualization of esophageal mucosa and the possibility to perform biopsies for histological examination, but its high cost and invasiveness limit the broad application of this technique and require the development of new preventive tools. Accordingly, a minimally invasive non-endoscopic device, the cytosponge-trefoil factor 3 (TFF3), has been recently proposed for the collection of esophageal cells in order to screen GERD patients who warrant endoscopy to diagnose BE [50]. The cytosponge is coupled with TFF3, an immunohistochemical biomarker for Barrett’s epithelium within a capsule attached to a string that must be swallowed and, when gelatin dissolves inside the stomach, the sponge is pulled up along the esophagus for cell collection. In the study by Ross-Innes CS et al. on a large sample of GERD patients, the sensitivity of this new device was 73.3% and the specificity 93.8%. More recently, Fitzgerald et al. [51] published in Lancet a new study in which the number of GERD patients diagnosed with BE was significantly greater with the cytosponge than with traditional endoscopy (140 vs. 13), and therefore this method seems to highly reliable for BE screening and should be preferred because it is simpler, mini-invasive, more comfortable, cheaper and more accurate than endoscopy with biopsy [51,52].

### 4.1. Esophageal Squamous Carcinoma

In Western countries, where the incidence of ESCC is relatively low, screening of the asymptomatic average-risk population is untenable. Individuals with high alcohol consumption and a long history of cigarette smoking are currently not included in the high-risk group that is expected to undergo endoscopic surveillance for ESCC.

At the moment, different groups of high-risk patients for ESCC have been identified and are represented by subjects who underwent a curative treatment for head and neck cancer and those affected by caustic injury, tylosis, or achalasia [51,53]. However, the efficacy, cost-effectiveness and time intervals of endoscopic surveillance are still matter of debate, given the limited evidence in this direction.

In order to perform a screening of ESCC, the application of narrow-band imaging (NBI) endoscopy and chromoendoscopy with Lugol solution staining has been proven useful. In fact, when dysplastic or cancerous changes of squamous epithelium develop, the contents of intracellular glycogen will decrease, and they will become Lugol-voided lesions (LVLs) [54,55]. However, many researchers have been studying and developing some new techniques, such as autofluorescence imaging (AFI), confocal laser endomicroscopy, endo-cytoscopy, and optical coherence tomography [56].

Patients with previous head and neck cancer are at higher risk of developing synchronous or metachronous ESCC, mainly due to the phenomenon known as “field cancerization”, which is related to exposure to the same risk factors (i.e., alcohol intake and smoking). The prevalence of ESCC in patients with head and neck cancer was found to range from 2.3% to 28%, while the risk of developing a synchronous or metachronous ESCC is highest in patients with hypopharyngeal and oropharyngeal cancers, followed by oral cavity, laryngeal and nasopharyngeal cancers [57]. Additionally, when secondary ESCC develops in patients with prior head and neck cancer, its prognosis is poor. These patients probably receive a late diagnosis of ESCC because they present persistent dysphagia or odynophagia after radiotherapy or surgery for the primitive cancer. Some evidence shows that an endoscopic screening to detect precancerous lesions and ESCC at an earlier stage is feasible and useful. The proposed and recommended endoscopy surveillance protocols usually start after curative treatment for head and neck cancer and continue every 6 months for 3–10 years to annual examinations [54]. The suggested surveillance method is Lugol chromoendoscopy or virtual chromoendoscopy.

ESCC can occur in the long-term in patients with previous caustic ingestion of either acid or alkali substances. The risk of ESCC in patients who had caustic injuries is 1000–3000 times higher than in the general population [57], and cancer usually develops 10–40 years after caustic injury [58,59]. Although there are no data about the effectiveness of endoscopic surveillance on this group of patients, some authors suggest performing endoscopic surveillance with white-light gastroscopy starting 10–20 years after the injury, with a minimum interval of 3 years [54].

ESCC can also develop in patients affected by achalasia, usually 10–15 years after diagnosis or 20–25 years from the beginning of symptoms [60]. The need for endoscopic surveillance in such a population is still debated, as studies so-far performed failed to demonstrate both the cost-effectiveness and the detection rate of early ESCC [61]. There is conflicting evidence on routine endoscopic surveillance for patients with achalasia. Some scientific societies conclude that there are insufficient data available, whereas some experts support surveillance with white-light endoscopy every three years in patients who received the diagnosis 10–15 years prior [51].

### 4.2. Esophageal Adenocarcinoma

#### 4.2.1. Endoscopic Screening

In literature, a large number of studies show that patients with chronic GERD symptoms benefit from endoscopic screening for BE because of the clear association between GERD, BE and EAC. Nevertheless, the cost-effectiveness analysis of endoscopic surveillance of BE is still undetermined. In fact, not only is the prevalence of GERD symptoms among the general population very high, and at least 40% of patients with BE do not have symptoms of GERD, but also no definitive data are available on whether endoscopic screening for BE is actually associated with a reduction in cancer-related mortality. Therefore, endoscopic screening in the GERD population is not routinely recommended [15].

The American College of Gastroenterology (ACG)’s clinical guidelines on BE report that screening for this disease may be considered in men that present chronic (>5 years) and/or frequent (weekly or more) symptoms of GER (heartburn or acid regurgitation), along with two or more risk factors for BE or EAC. These risk factors include: age > 50 years, Caucasian race, presence of central obesity (waist circumference >102 cm or waist-hip ratio >0.9), current or past history of smoking, as well as a confirmed family history of BE or EAC (in a first-degree relative) [62].

Considering that women with chronic GERD symptoms are subject to a substantially lower risk of EAC compared with men, screening for BE in the former is not recommended. However, screening should be considered in individual cases, as determined by the presence of the above-mentioned multiple risk factors for BE or EAC [62]. If the initial endoscopic evaluation is negative for BE, repeating this examination overtime is not recommended. If endoscopy reveals moderate or severe esophagitis (Los Angeles Classification grades B, C and D), repeating endoscopic assessment after PPI therapy for 8–12 weeks is recommended in order to ensure healing of esophagitis and to exclude the presence of underlying BE.

#### 4.2.2. Chemoprevention of Barrett’s Esophagus

Main treatments associated with secondary prevention for esophageal adenocarcinoma (Table 3).

The clinical guidelines reported above recommend that patients with BE should receive once-daily PPI therapy. Administering a twice-daily dose of PPI is not recommended unless the patients present scant control of reflux symptoms or erosive esophagitis [62]. Currently, several cohort studies report that subjects with BE with maintenance therapy with PPI present a decreased risk of degeneration into neoplastic BE compared with those either without a maintenance therapy or taking H2RA therapy [63,64,65,66]. A multicenter prospective cohort study demonstrated that PPI use was associated with a 75% reduction in the risk of neoplastic progression in patients with BE [62]. Furthermore, a later meta-analysis of observational studies concluded that the use of PPIs is associated with a decreased risk of esophageal adenocarcinoma and/or BE with high dysplasia in BE patients [67]. It is noteworthy that the risk profile of these medications is favorable in most patients [68,69], and the cost of this class of drugs has been substantially lower in recent years, as generic formulations of PPIs are available. These factors seem to justify the use of PPIs in BE populations, even in those without GERD symptoms [62].

According to ACG guidelines [62], aspirin or NSAIDs should not be routinely prescribed to patients with BE as an antineoplastic strategy. Similarly, other putative chemo-preventive agents have not yet demonstrated effectiveness, and thus they should not be administered routinely. However, ASA and NSAIDs have shown how the potential of inhibiting several pathways is crucial in oncogenesis, while some epidemiological studies revealed the chemo-preventive effect of ASA in the transition from BE to EAC [70,71,72,73,74,75,76,77,78,79,80]. The use of these chemo-preventive agents, due to their well-known adverse effects such as cerebral and GI hemorrhage, is not justified also considering that the estimated risk of progression in patients with non-dysplastic BE is low (0.1–0.5%/year). However, a very large (2557 patients) prospective randomized controlled trial in UK [7,81] has investigated the chemo-preventive effects of PPIs, both alone and in combination with aspirin (AspECT), and the results of this study showed that high-dose esomeprazole (40 mg twice-daily) and aspirin 300 mg or 325 mg in combination, significantly and safely improved outcomes in patients with BE after 8–9 years of follow-up. These authors stated that PPIs could also reduce the upper gastrointestinal bleeding associated with aspirin, whilst the benefits of this latter drug remain. So, although ACG guidelines do not justify the routine use of ASA or other NSAIDs in the chemoprevention of BE, the results of the AspECT study suggest that a revision of the above recommendations is warranted.

Statins are drugs used for primary and secondary prevention of cardiovascular diseases, and studies have suggested that they may have also a role in the chemoprevention of BE [82,83,84,85,86,87,88,89]. In particular, in-vitro studies on EAC cell lines have shown that statins have anti-proliferative, pro-apoptotic, anti-angiogenic and immuno-modulatory effects, thus preventing cancer development and growth [90,91,92]. Furthermore, a meta-analysis published in 2013 evaluated existing randomized controlled trials and observational studies about the association between statins and the risk of EAC or progression of dysplasia in patients with BE [93]. According to this study, the use of statins is associated with a 41% reduction in the risk of neoplastic progression, after adjusting for potential confounders (i.e., use of NSAIDs/aspirin and baseline BE segment length and dysplasia grade). In addition, the meta-analysis of the two studies that assessed the combined effect of statins and NSAIDs/aspirin use on development of EAC in BE has demonstrated a reduction of 72% in EAC incidence. Considering the above results, it seems useful to conduct more chemo-preventive trials that better evaluate the effects of statins in BE.

Obesity and, in particular, central adiposity are risk factors associated with BE and EAC. In fact, the activation of the insulin/IGF pathway is associated with BE-mediated carcinogenesis [94]. A double-blind, randomized, controlled prospective chemoprevention trial of metformin in BE patients was conducted and published in 2015 [95]. This study showed that metformin 2000 mg daily in BE patients on PPI, without considering insulin-resistance or diabetes mellitus II, although tolerable and safe, was not effective in altering proliferation or apoptosis pathways in non-dysplastic BE. It will be necessary to conduct more studies to elucidate this aspect and perhaps consider alternate carcinogenic pathways involving adiponectin and/or leptin.

Anti-reflux surgery (ARS) might be a preventive measure against transition from BE to EAC. Although in the past two years several systematic reviews and meta-analyses have been published about the possible preventive role of ARS on the neoplastic progression of BE, none of them have found any significant effect on the development of EAC compared with medical treatment [96,97,98]. Nevertheless, a recent systematic review and meta-analysis published in 2016 has provided some evidence that, in patients with BE, ARS may prevent EAC better than medical therapy [99]. This could be explained by the fact that medical treatments decrease the acidity of refluxate but do not prevent reflux per se. A recent 5-year follow-up of a randomized clinical trial that used pH-measurements also showed how, in GERD patients, surgery leads to lower levels of abnormal acid reflux in the esophagus compared with medication [100]. In addition, compared with PPI therapy, fundoplication is not dependent on dosage or therapy adherence. However, according to this meta-analysis, the risk of EAC seems to remain higher in patients after ARS compared with the background population. This may be due to the particularly high severity of GERD in patients selected for ARS, where DNA is already damaged, leaving the operated patients to a long-term increased risk of EAC compared with the population at large. Furthermore, some of the patients undergoing ARS will have recurrence of GERD, thus further increasing the risk of EAC compared with the background population [99].

In conclusion, more data from larger studies with longer follow-up periods are required to determine the actual role of ARS in the control of the progression from BE to EAC.

#### 4.2.3. Barrett’s Treatment

During the last decade, many studies have been published about the potential treatment of BE and the control of its progression from non-dysplastic BE to LGD, HGD and, subsequently, EAC. Nowadays, the most debated point regards whether LGD must undergo endoscopic ablation or endoscopic surveillance.

ACG guidelines about management of BE report that endoscopic eradication therapy is the preferred procedure for patients with BE and confirmed HGD [62]. Although endoscopic surveillance every 12 months is a good compromise in patients with LGD and without life-limiting comorbidity, endoscopic therapy should be the preferred modality. In contrast, ablative therapy cannot be recommended in patients with non-dysplastic BE due to the very limited evidence of neoplastic progression, along with the costs associated with the procedure and the risk of complications linked to ablative therapy. It is unclear whether it is recommended to perform such therapy in subjects considered to have a higher life-time risk of cancer, such as those with familial BE/EAC and young patients with long segments of BE [62]. In patients with BE and confirmed LGD, recent data show that ablative therapy results in a statistically and clinically significant reduction in the risk of degeneration to the combined end point of HGD or EAC, or to EAC alone. In the AIM-Dysplasia trial, patients with LGD were randomized to radiofrequency ablation (RFA) or to sham ablation and followed-up for one year [101]. Progression to HGD at twelve months was 5% in the RFA group compared with 14% in the sham-treated group, and this difference was significant. Five years later, the European SURF study randomized patients to RFA or endoscopic surveillance [102], and at the end of the three-year follow-up, 1.5% of treated patients developed high-grade dysplasia or EAC compared with 26.5% in the surveillance group. Lastly, a multicenter cohort study compared the efficacy of RFA against endoscopic surveillance in patients with LGD and showed a rate of progression to HGD or EAC that was significantly lower in the RFA group [103]. The above three studies show that ablative treatment is a better choice than endoscopic surveillance in patients with LGD [104]. Nonetheless, negative points about a more widespread endoscopic ablation of LGD are present in these studies. In the AIM and SURF studies, respectively, 22% and 28% patients in the surveillance arm experienced resolution of dysplasia during follow-up and have thus undergone unnecessary ablation. Other drawbacks of RFA include the cost and the need for multiple upper endoscopies even after complete ablation.

**Table 3:** Main treatments associated to secondary prevention for esophageal adenocarcinoma.

Drug	Mechanism of Action	Type of Study	Main Findings and Reported Relative Risks or Odds Ratios (OR),	Recommendations
Statins	Vitro and animal studies: ✓ Anti-inflammatory and antineoplastic effects through both HMG-CoA reductase-dependent and HMG-CoA reductase-independent pathways. ✓ Inhibition of several downstream products of the mevalonate pathway, including the generation of isoprenoids. This prevents post-translational prenylation of the small signaling G-proteins of the Ras/Rho/ Rac superfamily. ✓ Pro-apoptotic effects through regulation of Rho and RAF mitogen activated the protein kinase 1-extracellular regulated kinase (MEK-ERK) pathway through a HMG-CoA reductase dependent mechanism. ✓ Inhibition of the activation of the proteosome pathway, limiting the breakdown of cyclin-dependent kinase inhibitors p21 and p27, thus allowing these molecules to exert their growth-inhibitory effects.	Observational [66,83,84,85,86,87,88,90] Meta-analysis [82,89,93]	A significant reduction in the risk of esophageal cancer among patients who took statins (adjusted OR, 0.72; 95% CI, 0.60–0.86). In BE patients, statins were associated with a significant (41%) decrease in the risk of EAC (adjusted OR, 0.59; 95% CI, 0.45–0.78) [93] Regular statin use was associated with a significantly lower incidence of Barrett’s esophagus [adjusted OR 0.62 (95% confidence intervals 0.37–0.93)]. This effect was more marked in combined statin plus aspirin users [adjusted OR 0.43 (95% CI 0.21–0.89)] [82]. Statin use was significantly associated with a reduced risk of Barrett’s esophagus [pooled adjusted OR 0.63 (95% CI 0.51–0.77)] [82]	Statin use is not recommended as a chemo-preventive agent
Proton Pump Inhibitors Use	✓ Irreversible block of the H+/K+ ATPase enzyme that is necessary for the production of cloridric acid by the gastric parietal cells reducing acid esophageal exposure time.	Observational [63,64,65] Meta-analysis [67]	The use of PPIs included in the study or during the follow-up period reduced the risk of neoplastic progression (Hazard ratio, 0.41; 95% confidence interval, 0.18–0.93 and hazard ratio, 0.21; 95% confidence interval, 0.07–0.66) [64]	Once daily PPI therapy have to be assumed
NSAID and aspirin	✓ Mechanism of potential risk reduction is unknown but may be related to the inhibition of the cyclooxygenase-2 enzyme, which is induced early in the development of numerous tumors, including esophageal carcinomas. Its activity may contribute to cancer growth through several mechanisms, including increasing cells’ longevity via inhibition of apoptosis, stimulation of angiogenesis, or other effects on the cell cycle.	Observational [71,72,73,74,75,76,77,78,79] Meta-analysis [80]	Statistical pooling showed a protective association between any use of aspirin/NSAID and esophageal cancer (OR 0.57; 95% CI 0.47– 0.71). Both intermittent (OR 0.82; CI, 0.67–0.99) and frequent medication use were protective (OR 0.54; CI, 0.43–0.67), with greater protection with more frequent use. Stratified by medication type, aspirin use was protective (OR 0.5; CI, 0.38–0.66), and NSAIDs had a borderline protective association (OR 0.75; CI 0.54–1.0). Any use was protective against both esophageal adenocarcinoma (OR 0.67; CI, 0.51–0.87) and squamous cell carcinoma (OR 0.58; CI, 0.43–0.78) [80]	Aspirin and NSAIDs are not recommended as chemo-preventive agents
Metformin	✓ Protection against obesity-associated cancers. ✓ Reduces serum insulin levels inhibiting cell growth directly because insulin has been associated to cellular proliferation and inhibits apoptosis.	Observational [95]	-	Metformin is not recommended as a chemo-preventive agent
Anti-Reflux Surgery	✓ Reinforces the anti-reflux barrier at the esophago-gastric junction level by creating a wrap around it. ✓ Reduces the reflux burden and eliminates the main risk factor associated to the development and disease progression of Barrett’s esophagus	Observational [96] RCT [100] Meta-analysis [97,98,99]	In patients with Barrett’s esophagus, the corresponding IRR was 0.46 (95% CI 0.20–1.08) and 0.26 (95% CI 0.09–0.79) when restricted to publications after 2000 [99]	Anti-reflux surgery may prevent EAC better than medical therapy in patients with Barrett’s esophagus
Barrett’s Treatments	✓ Endoscopic treatments of Barrett’s esophagus (radiofrequency and cryoablation alone or with esophageal mucosectomy) permit us to eradicate the intestinal metaplasia and dysplasia, reducing the disease progression of Barrett’s esophagus	Observational [101,103,105] RCT [102] Meta-analysis [104]	The progression of BE-LGD to either HGD or EAC was significantly lower in patients treated with RFA compared with endoscopic surveillance (OR: 0.17, 95% CI: 0.04–0.65, *p* = 0.01). The progression to HGD alone was significantly lower in patients treated with RFA vs. endoscopic surveillance (OR: 0.23, 95% CI: 0.08–0.61, *p*= 0.003). Progression to EAC alone was numerically lower in RFA compared with endoscopic surveillance without statistical significance (OR: 0.44, 95% CI: 0.17–1.16, *p* = 0.09) [104]	It is recommended endoscopic eradication therapy with RFA, PDT or EMR in Barrett esophagus with high grade dysplasia

PPI: pomp proton inhibitors; BE: Barrett Esophagus; RFA: radiofrequency ablation; PDT: photodynamic therapy; EMR: endoscopic mucosal resection; LGD: low grade dysplasia; HGD: high grade dysplasia; EAC: esophageal adenocarcinoma.

According to ACG guidelines [62], in case of the detection of mucosal irregularity, including nodularity, ulceration, or flat but irregular mucosal contour, endoscopic mucosal resection (EMR) or endoscopic submucosal dissection (ESD) should be performed. The histological findings of these two endoscopic resections determine subsequent management of patients. In case of a history of non-dysplastic BE, surveillance endoscopy can be pursued if EMR does not demonstrate evidence of dysplasia. On the other hand, if EMR demonstrates presence of LGD or HGD and patients underwent complete resection of lesions, endoscopic ablative therapy should follow the endoscopic resection.

## 5. Tertiary Prevention

### 5.1. Surveillance for ESCC

Surveillance after EMR or ESD for ESCC aims to detect and treat recurrences, metachronous ESCC, and second primary cancers (such as head, neck, gastric, lung and colorectal cancer) early (Table 4).

Local recurrence after EMR or ESD usually occurs within one year after initial treatment, and if it develops after two to three years, then a long-term follow-up is required [105]. Lugol chromoendoscopy is the more frequently used technique to detect local recurrence. The proposed long-term surveillance protocol includes endoscopy at 6-month or at 3-month intervals for up to 6 months to 1 year after resection [54]. In the presence of multiple LVLs, EMR is associated with a higher risk of local recurrence than ESD [106].

After the endoscopic resection of ESCC, patients are prone to a high incidence of metachronous ESCC (10–15%), which can develop at any time after treatment. As the presence of LVLs is considered the most important risk factor for metachronous ESCC [107], a strict long-term follow-up with Lugol chromoendoscopy is essential. The proposed long-term surveillance protocol includes endoscopy at 6-month intervals or at 3-month intervals for 6 months up to 1 year after resection [54].

A national registry established by the Japan Esophageal Society found that secondary tumors develop in 20% patients with ESCC [108]. In these patients, regular follow-up of the head, neck, esophagus and stomach by upper endoscopy is essential. Cancer screening should include examination of the head and neck region by an otolaryngologist, chest radiography or CT, as well as a colorectal examination. However, standardized protocols for follow-up have yet to be established. The currently proposed endoscopic surveillance protocols, using Lugol chromoendoscopy or virtual chromoendoscopy, usually start after curative treatment for head and neck cancer and continue every 6 months for 3–10 years and then proceed to annual examinations [54].

### 5.2. Incidence of ESCC after Surgery of Achalasia

We have already said that ESCC occurs at higher frequency in patients with esophageal achalasia, but it has also been reported that the incidence rate of this cancer is high during follow-up after surgical treatment (2.9%). In fact, it can occur many years after the initial detection or treatment of achalasia (11–15 years) [109].

It is still debated whether endoscopic surveillance after curative surgery for achalasia should be performed, and very few data can be found in the medical literature on this topic. In a recent study, 32 patients who were treated for esophageal achalasia underwent a long-term and annual upper GI endoscopy follow-up [109]. ESCC was detected in 6 patients, and the average duration of follow-up until cancer after surgery was 14.3 years (range 5–40 years). Five of these patients had early cancer revealed after the annual endoscopy and were all treated with endoscopic resection. However, data about the best time interval of follow-up after surgery are lacking. The same study suggests that the potential for malignant transformation persists even when improving the passage symptoms after surgery, as patients, who subsequently developed ESCC, had a clinical remission of achalasia after operation.

### 5.3. Endoscopic Surveillance for BE

In order to detect EAC at an early and curable stage, when endoscopic treatment is still feasible and associated with good survival, endoscopic surveillance after diagnosis of BE is recommended (Table 4) with different surveillance intervals depending on the presence and grade of dysplasia [62].

In BE patients without dysplasia, endoscopic surveillance should take place at intervals of 3–5 years. As illustrated above, for patients with confirmed LGD and without life-limiting comorbidity, endoscopic therapy is considered the preferred treatment modality, even though endoscopic surveillance every 12 months is an acceptable alternative. Patients with BE and confirmed HGD should be treated with endoscopic therapy unless they have life-limiting comorbidity. In contrast, after optimization of acid suppressive therapies for 3–6 months, endoscopy should be carried out in patients with indefinite evidence for dysplasia. If indefinite findings for dysplasia are confirmed on the repeated examination, a surveillance interval of 12 months is recommended [62].

High-definition/high-resolution white light endoscopy (HD-WLE) should be preferred to standard-definition white light endoscopy for the detection of dysplastic lesions during surveillance of BE [110].

In recent years, a wide variety of advanced imaging techniques has been studied to allow for correct inspection of BE segments. Narrow band imaging (NBI) uses spectral narrow band filters that allow for visualization of esophageal mucosal and vascular patterns, similar to chromoendoscopy but without the time expense for dye. A randomized controlled trial has shown no difference in the detected number of patients with dysplasia or EAC, using NBI compared with HD-WLE. However, NBI-targeted biopsies can have the same detection rate of BE and dysplasia as HD-WLE with the Seattle protocol, while requiring fewer biopsies [111]. The data collected so far and the high cost of this technique do not justify the use of NBI with the only goal of detecting HGD or EAC. Electronic chromoendoscopy increases the diagnostic yield of dysplasia and EAC in patients with BE [112], and this is the only advanced imaging technique recommended for endoscopic surveillance of BE at this time [62].

According to the ACG Guidelines about the management of BE, endoscopic surveillance should employ four-quadrant biopsies at 2 cm intervals in patients with non-dysplastic BE, while biopsies at 1 cm intervals should be performed in patients with BE with dysplasia. In addition, mucosal abnormalities such as ulceration, erosion, plaque, nodule, stricture or other luminal irregularity in BE segments should be sampled separately, preferably with EMR [62]. The detection of dysplasia during endoscopic surveillance of BE influences the clinical and therapeutic management of patients. However, there is considerable interobserver variability in the interpretation of dysplasia, particularly for indefinite findings for dysplasia and LGD [113,114]. Two recent studies have shown that LGD in BE is an over-diagnosed entity in the community and suggest that patients affected by LGD should undergo an expert pathology review to be down staged [115,116]. It is also likely that community pathologists are subject to marked interobserver variation in the interpretation of both HGD and LGD [116]. As a result, according to ACG guidelines, BE patients with dysplasia of any grade are recommended to receive a review by two different pathologists, of which one should have specialized expertise in gastrointestinal pathology, in order to avoid possible interobserver variability in the interpretation of dysplasia [62].

## 6. Conclusions

In conclusion, the primary means of prevention for EAC and ESCC is to identify the risk factors and, if present, eliminate them. The secondary prevention consists of the detection of precancerous and early cancerous lesions when curative endoscopic treatment is still possible. The tertiary means of prevention is to allocate endoscopic resources to subjects in whom precancerous or early cancer conditions have already been identified or treated. In particular, we believe that in the future, screening programs for ESCC in high-risk patients and surveillance programs in patients with ESCC that has already been treated need to be defined.

As for patients with BE, although chemo-preventive trials considering statins and metformin in order to prevent progression to EAC are needed, a recent, very large study has shown that high-dose esomeprazole in combination with aspirin is able to significantly and safely improve the outcomes in BE, and this suggests a revision of current guidelines that do not justify the use of aspirin or NSAIDs in the prevention of EAC. In high-risk patient groups for ESCC or EAC, advanced imaging techniques must be applied in order to perform screening and surveillance, but their cost-effectiveness and efficacy must be accurately evaluated.

## Figures and Tables

**Table 1:** Risk factors for esophageal cancer.

Risk Factor	Squamous-Cell Carcinoma	Adenocarcinoma	Note
Tobacco use	+++	++	Tobacco contains carcinogens and promotes inflammation Tobacco and alcohol use are two factors that work synergistically.
Alcohol use	+++	−	Tobacco and alcohol use are two factors that work synergistically
Mutations of enzymes that metabolize alcohol			Genetic susceptibility, e.g., loci at PLCE1, C20orf54, ADH1B and ALDH2 coupled with alcohol consumption and smoking
Barrett’s esophagus	−	++++	
Weekly reflux symptoms	−	+++	
Obesity	−	++	It increases gastroesophageal reflux and inflammatory cytokines
Poverty	++	−	
Achalasia	+++	−	
Caustic injury to the esophagus	++++	−	
Non-epidermolytic palmoplantar keratoderma (tylosis)	++++	−	
Plummer-Vinson Syndrome	++++	−	
History of head and neck cancer	++++	−	
History of breast cancer treated with radiotherapy	+++	+++	
Frequent consumption of extremely hot beverages	+	−	
Prior use of beta-blockers, anticholinergic agents, or aminophylline	−	±	
HPV 16 and 18 in some areas		−	

A single plus sign indicates an increase in the risk by a factor of less than two, two plus signs an increase by a factor of two to four, three plus signs an increase by a factor of more than four to eight, four plus signs an increase by a factor of more than eight. The plus-minus sign indicates that conflicting result have been reported, and the dashes indicate that there is no proven risk. HPV = Human Papilloma Virus.

**Table 2:** Main lifestyle and clinical factors known to influence the development of esophageal cancer and related recommendations.

Lifestyle and Clinical Factors	Mechanism of Action	Type of Study	Main Findings and Reported Relative Risks (RR) or Odds Ratios (OR)	Recommendations
**Smoking**	✓ Tobacco smoke is known to contain polycyclic aromatic hydrocarbons, nitrosamines and many other carcinogens. ✓ Cigarette smoke is known to contain a large number of pro-oxidative substances and generates reactive oxygen species, which can initiate and promote carcinogenesis	Observational [24,25,26,27,31]	Tobacco smoking cessation < 10 y: OR, 0.82; 95% CI, 0.60–1.13 [31] Tobacco smoking cessation ≥10 y: OR, 0.71; 95% CI, 0.56–0.89 [31]	Abstinence from smoking. The synergic action with alcohol is important to know and to correct.
**Food**	✓ Hot beverages can cause recurrent thermal injury ✓ Fruit/vegetables intake have chemo-preventive effects for their high levels of micronutrients (including antioxidants), which can decrease DNA damage by scavenging for oxygen radicals. They contain flavones, which inhibit the cell process associated with carcinogenesis, possibly through their effects on focal adhesion kinase and metalloproteinases. ✓ Fiber might also partially mediate the associations found for fruits and vegetables. ✓ Processed meats may have a high nitrate content, which can initiate and promote carcinogenesis	Observational [25] Meta-analysis [30]	The overall pooled RR of EC and the confidence intervals for the groups with the highest versus the lowest levels of intake were as follows: 0.99 (95% CI: 0.85–1.15) for total meat; 1.40 (95%CI: 1.09–1.81) for red meat; 1.41 (95%CI: 1.13–1.76) for processed meat; 0.87 (95%CI: 0.60–1.24) for poultry; 0.80 (95%CI: 0.64–1.00) for fish [30]	Avoidance of meat, processed food intake, hot beverages High fruit/vegetable intake Abstinence from betel quid chewing
**Alcohol**	✓ Acetyl-aldehyde may cause cellular damage and have a carcinogenic effect. ✓ A commonly accepted interpretation of the synergy between ethanol and tobacco smoke is that ethanol dissolves and facilitates the transport of tobacco carcinogens to cells, making them more susceptible to carcinogenesis.	Observational [21,24,25,26,27]		Abstinence from alcohol consumption. It is important to correct the synergic action with tobacco

**Table 4:** Suggested surveillance methods of patients with Barrett’s esophagus and squamous-cell carcinoma as recommended by different American and European scientific societies.

Cancer	Suggested Surveillance Methods				Suggested Surveillance Intervals			
	ESGE	AGA	ASGE	BSG	ESGE	AGA	ASGE	BSG
**Esophageal adenocarcinoma**	Endoscopy or ultrathin nasal endoscopy with biopsy	High-definition white-light endoscopy and 4-quadrant biopsy specimens taken every 1–2 cm of Barrett’s mucosa, depending on the degree of dysplasia	Endoscopy with biopsy	Endoscopy with biopsy/HRE in BE. Adherence to a quadrant, 2 cm biopsy protocol in addition to sampling any visible lesions. Expert HRE should be carried out in all Barrett patients with biopsy-detected HGD	No dysplasia: every 5 years LGD: every 3 years in long-segment BE	No dysplasia: every 3–5 years LGD: every 6–12 months HGD: in the absence of eradication therapy every 3 months	No dysplasia: Consider no surveillance. If surveillance is elected, every 3 to 5 years LGD: after the repeat endoscopy in 6 months to confirm LGD, every year. HGD: every 3 months in selected patients.	No dysplasia: after the repeat endoscopy to confirm the diagnosis, patients with BE shorter than 3 cm and IM should receive endoscopic surveillance every 3–5 years. If repeat endoscopy confirms the absence of IM, discharge from surveillance is encouraged Patients with segments of 3 cm or longer should receive surveillance every 2–3 years. LGD: every 6 months
**Esophageal squamous-cell carcinoma**	Endoscopy with biopsy	Endoscopy with biopsy	Endoscopy with biopsy	Full assessment with enhanced imaging and/or Lugol’s chromo-endoscopy is required	Surveillance intervals vary from 2 to 5 years	The interval for surveillance of these patients has not been established, but yearly investigations would seem to be reasonable	Tylosis: screening at 30 years of age or at the recognition of the disease and then every 1 to 3 years. Caustic injury: screening should begin approximately 10 to 20 years after the insult, and then every 2 to 3 years	

ESGE = European Society of Gastrointestinal Endoscopy; AGA = American Gastroenterological Association; ASGE = American Society for Gastrointestinal Endoscopy; BSG = British Society of Gastroenterology; BE = Barrett Esophagus; HRE = High-resolution endoscopy; HGD = High-grade dysplasia; ER = Endoscopic Resection; LGD = low grade dysplasia; IM = intestinal metaplasia.

## Data Availability

Data is contained within the article.
